# Novel Use of an Aseptically Processed Amnion-Chorion Placental Allograft to Complement Wound Closure in High-Risk Sternotomy Patients: Clinical Safety and Outcomes

**DOI:** 10.7759/cureus.73322

**Published:** 2024-11-09

**Authors:** Zain Khalpey, Ujjawal Kumar, Zacharya I Khalpey, Pamela Hitscherich, Evangelia Chnari, Marc Long

**Affiliations:** 1 Department of Cardiothoracic Surgery, HonorHealth, Scottsdale, USA; 2 Khalpey AI Lab, Applied & Translational AI Research Institute (ATARI), Scottsdale, USA; 3 School of Clinical Medicine, University of Cambridge, Cambridge, GBR; 4 Department of Research, MTF Biologics, Edison, USA

**Keywords:** cardiovascular surgery, frailty, obesity, placental allograft, sternotomy

## Abstract

Objectives

Wound dehiscence is defined as the partial or complete separation of the layers of a surgical wound. Wound dehiscence and infections are of significant concern in the field of surgery as they can lead to a range of complications, including infection, delayed healing, increased healthcare costs, and patient discomfort. For patients at high risk of sternal wound dehiscence and infection, optimization of wound closure is critical. Novel technologies are increasingly being developed to optimize wound closure following median sternotomy for cardiac surgery. Aseptically processed amnion-chorion placental allografts (aACPA) are one such example. Placental allografts maintain the inherent growth factors and matrix proteins native to the tissue, all of which are known in the literature for their roles within the natural closure of wounds

Methods

Twenty-six patients who underwent cardiac surgery requiring a median sternotomy at a single center undertaken by a single surgeon were included in this study. All patients included were deemed high-risk for sternal complications and had at least one sternal risk factor. Before closure, 160 mg of aACPA was added to the sternotomy wound to support wound repair. Data were collected for rates of sternal complications, as well as general demographics and past medical history of patients included in this study, and appropriate analyses were carried out.

Results

At their 14- and 30-day follow-up visits, none of the patients had experienced sternal wound dehiscence or infection, with their sternotomy wounds showing excellent signs of normal wound closure. A comprehensive sternal pain evaluation was carried out, which elicited no significant pain in any patients, a sign that sternal closure was successful and stable. The addition of the aACPA into our clinical practice has also contributed to no longer requiring postoperative chest stabilization adjuncts, resulting in significant financial and resource savings for our group.

Conclusions

In this study, the amnion-chorion placental allograft showed promise as an effective solution to support sternal wound closure in high-risk patients. Its inherent growth factors and ECM (extracellular matrix) may directly address the specific challenges faced by these high-risk individuals. This innovative treatment offers a novel and advanced approach to support wound closure in patient populations that are particularly vulnerable to complications.

## Introduction

Despite advances in minimally invasive approaches, median sternotomy remains the most common approach for cardiac surgery, providing optimal access to the heart and great vessels [1]. However, this technique is associated with significant risks, particularly in high-risk patient populations. Sternal dehiscence and deep sternal wound infections are among the most devastating complications of cardiac surgery, leading to delayed recovery, prolonged inpatient stays, increased resource utilization, and poor prognosis. Indeed, sternal complications are the primary indication for around 20% of 30-day hospital readmission rates [2], indicating not only their impact on morbidity and mortality but also on resource costs to hospitals and healthcare systems.

Despite current preventive strategies, including prophylactic antibiotic treatment, antibiotic impregnation of the surgical site, advanced sternal closure solutions, and negative pressure dressings, the complication rate remains substantial. For high-risk patients, including those with obesity, diabetes, frailty, a history of repeated surgeries, or immunosuppression, the sternal complication rate can be as high as 10% [3]. Even with optimal care, a 3-5% complication rate persisted across this patient group at our institution, leading us to consider adjunctive measures that could be implemented to improve outcomes for this high-risk patient population.

In recent years, regenerative medicine approaches have gained attention for their potential to enhance wound healing and reduce complications. Among these, amnion-chorion placental allografts (ACPA) have shown promise in various surgical applications. ACPAs are derived from placental tissue obtained from healthy, screened mothers and are known to promote tissue remodeling and wound closure while reducing fibrotic tissue formation [4]. The extracellular matrix components present in ACPA serve as a scaffold for cell migration and proliferation, while intrinsic cytokines and growth factors such as platelet-derived growth factor (PDGF), vascular endothelial growth factor (VEGF), and transforming growth factor-beta (TGF-β) provide a supportive environment for host cell proliferation, angiogenesis, and tissue remodeling [5].

This study investigates the potential of an aseptically processed ACPA (aACPA, Salera®, MTF Biologics, Edison, NJ), in supporting wound closure following median sternotomy in high-risk cardiac surgery patients. We hypothesize that the application of aACPA, as part of a comprehensive wound closure strategy, may reduce the incidence of sternal complications in this vulnerable patient population. By evaluating key outcomes such as intensive care unit length of stay, overall hospital length of stay, the incidence of sternal wound infections and dehiscence, and postoperative pain, we aim to assess the efficacy of this novel approach in addressing the persistent challenge of sternal wound complications in cardiac surgery.

## Materials and methods

This is a retrospective observational cohort study, comprised of patients who underwent cardiac surgery via median sternotomy at HonorHealth, Scottsdale, AZ, USA. Institutional Review Board ethical approval was granted for outcome analysis in this study (IRB-23-0025, October 29, 2023) and informed consent was obtained for all patients for the relevant surgical procedures as well as anonymized inclusion into this study. All methods of this study were conducted following the relevant regulations for working with human subjects and the Declaration of Helsinki [6]. All patients included were over the age of 18. Patients with previous sternal wound complications were excluded.

Sternal wound infections and dehiscence were defined as per the Society of Thoracic Surgeons’ criteria [7]. Heart failure was defined using American Heart Association (AHA) criteria for diastolic heart failure as a left ventricular ejection fraction (LVEF) less than or equal to 40% by transthoracic echocardiography [8]. Chronic kidney disease (CKD) was defined using an internationally conventional set of guidelines from KDIGO (Kidney Disease: Improving Global Outcomes) [9]. These criteria define chronic kidney disease as having a glomerular filtration rate of less than 60 ml/min/1.73m^2^ and albuminuria with an albumin-creatinine ratio greater than 3 mg/mmol [9].

Data collection

Demographic data, preoperative clinical characteristics, operative characteristics, and postoperative outcomes were collected from the institutional electronic health record system (Epic) [10]. Data collected was anonymized and stored on a secure server as per institutional information governance protocols for outcomes research data. Pre-operative data collected included key surgical risk scores (STS mortality and deep sternal wound infection risk scores) and data regarding risk factors for sternal complications such as prior sternotomy, chronic obstructive pulmonary disease (COPD), a smoking history, or long-term immunosuppressive medication use. Data was also collected for other comorbidities such as hypertension (HTN), obstructive sleep apnea (OSA), atrial fibrillation (AFib), and chronic kidney disease (CKD) as well as key risk scores such as CHA_2_DS_2_-VASc and HAS-BLED.

Operative technique and clinical protocol

**Figure 1:**
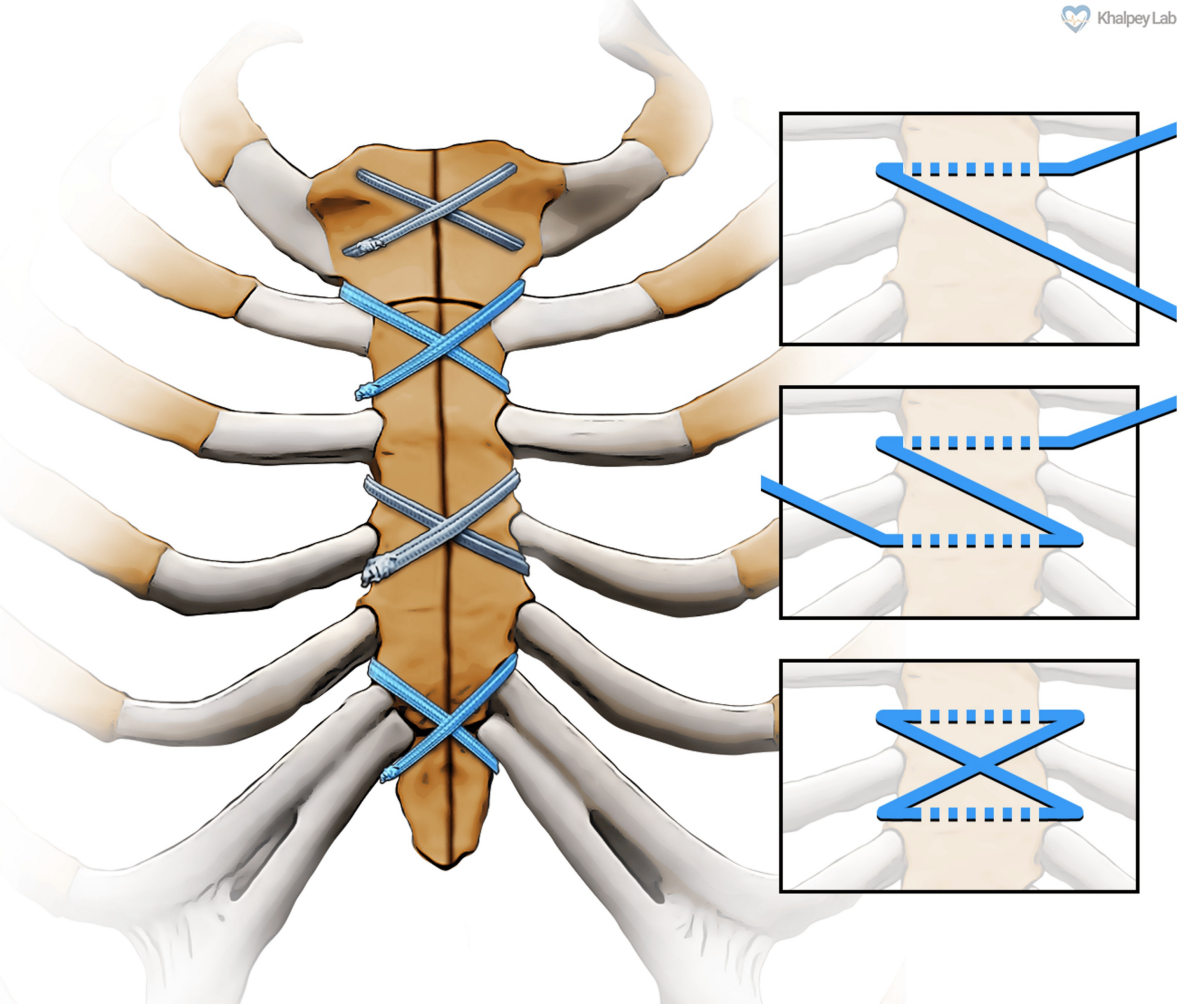
Suture tape sternal closure using the TigerTape sternal closure system. Figure credits: Mason Wiest and Ujjawal Kumar

After the main aspects of the surgical procedure were completed, attention was turned to sternal reapproximation. The sternum is closed using ultrahigh molecular weight polyethylene suture tapes (TigerTape, Arthrex Inc., Naples, FL; Figure 1). These are pre-soaked in vancomycin solution for five minutes before use. The TigerTape, black and white, and FiberTape, blue and white, measure 2 mm in width. In figure-of-eight patterns, four sutures are placed through the manubrium and through the sternal interspaces around the sternum. A tensioner is used to sequentially tighten each figure-of-eight with 60 to 80 lb of pressure and a half-hitch knot tied to lock the sutures.

**Figure 2:**
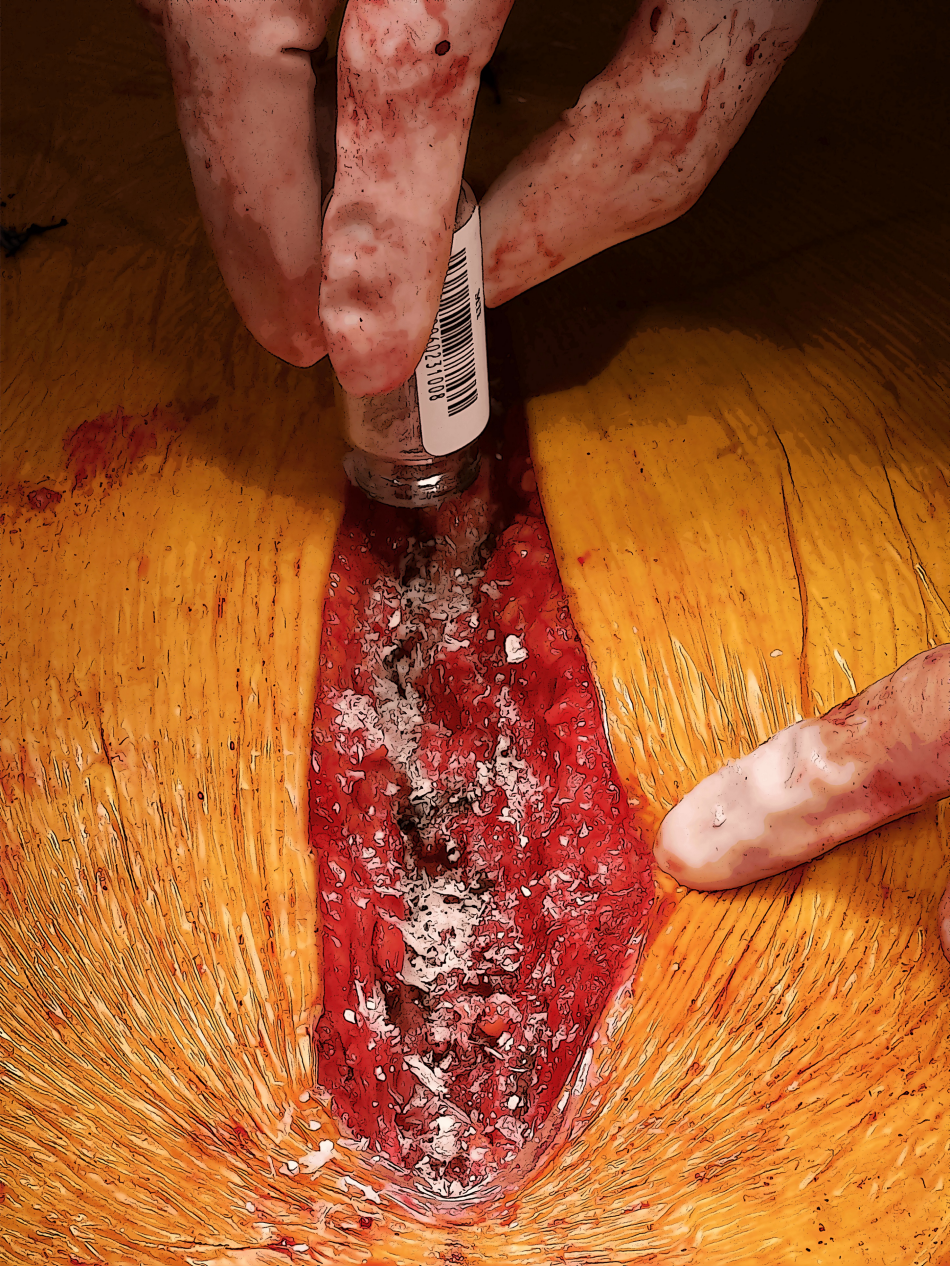
Median sternotomy showing the reapproximated sternum with the aACPA spread into the wound. Figure credits: Zain Khalpey, Ujjawal Kumar, and Mason Wiest

Next, wound lavage is carried out with a vancomycin solution, and 160mg of aACPA (aseptically processed, no terminal irradiation, amnion-chorion placental allograft, Salera®, MTF Biologics, Edison NJ) are added to the sternum and subcutaneous tissues before closure (Figure 2). Closure is achieved with 0, 2-0, and 4-0 Stratafix (Ethicon Inc., Cincinnati, OH). The wound is dressed with a JumpStart electroceutical dressing (Arthrex Inc., Naples, FL).

Follow-up and outcomes

Postoperatively, data was collected for the total ICU and hospital lengths of stay (LOS). Data was also collected on the incidence of key outcomes such as hospital death, sternal wound infection, and dehiscence. In line with our institutional protocols, postoperative follow-up of cardiac surgical patients was conducted two weeks and one month after the operation. These check-ups encompassed a thorough evaluation of multiple bodily systems and a review of prescribed medications. Pain was deemed significant if it severely impacted the patient's daily functioning and recovery process, or if a previously unused opioid was newly prescribed. Each follow-up session included a thorough wound inspection and pain assessment. To evaluate pain, the surgeon applied pressure to the breastbone and between the ribs. Furthermore, while the patient stood upright, the surgeon placed two fingers on the breastbone and asked the patient to rotate their upper body sideways to check for any pain response. Data was collected on any significant pain at 14- and 30-day postoperative follow-up appointments.

Statistical analyses

Data were summarized using descriptive statistics. For continuous variables, mean and standard deviation (SD) were presented for normally distributed data, with non-parametric data presented as a median and interquartile range. Categorical variables were presented as N (%). All statistical analyses were performed using R v4.4.1 (R Foundation, Vienna, Austria) [11] and GraphPad Prism v10.1.0 for macOS (GraphPad Software, Boston, MA, USA) [12]. A p-value less than 0.05 was considered significant as is conventional.

## Results

Patient characteristics

**Figure 3:**
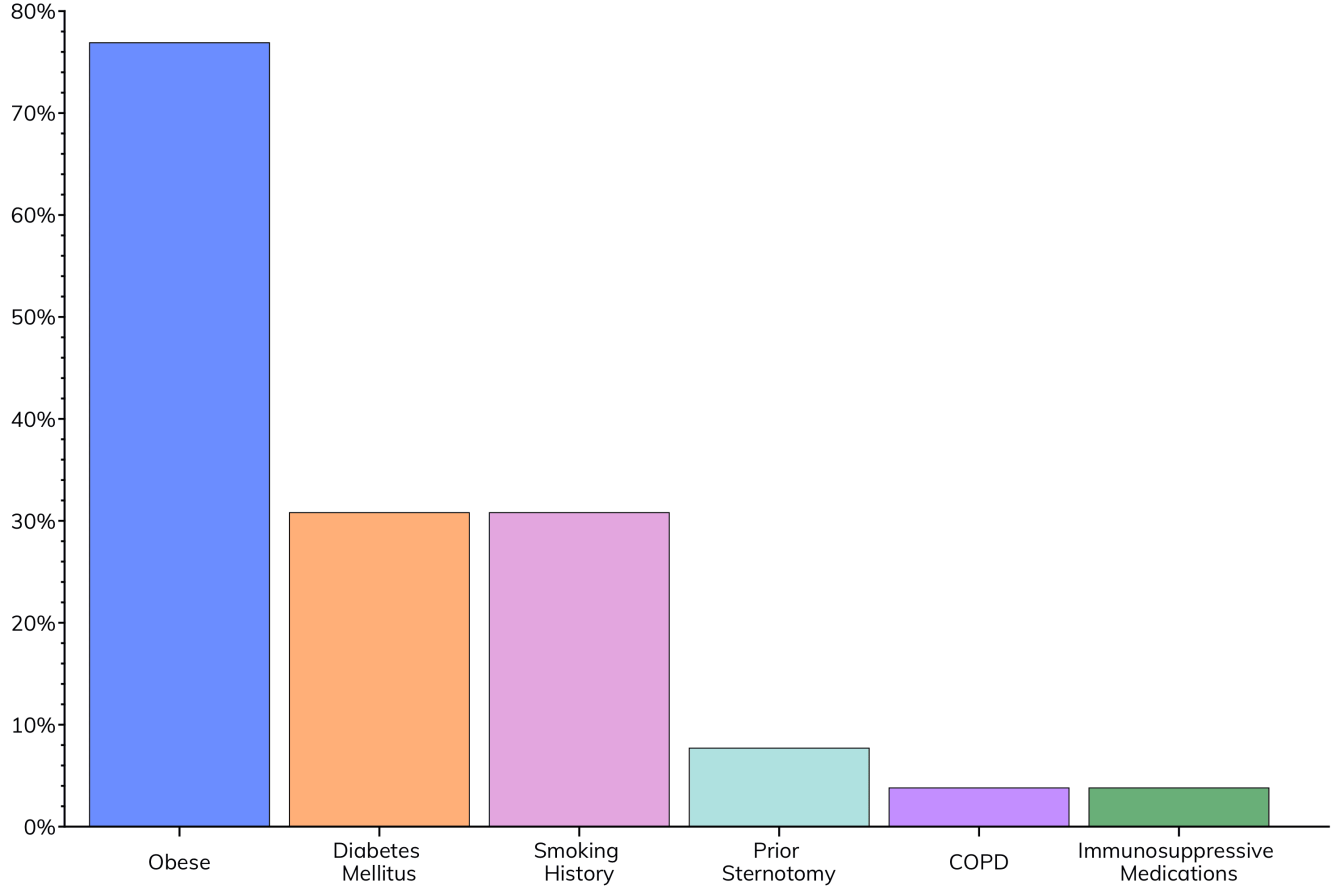
Prevalence of risk factors for sternal complications within the study population.

The study population consisted of 26 patients with various risk factors for sternal complications following cardiac surgery (Figure 3, Table 1). Obesity was the most prevalent risk factor, affecting 20 (76.9%) patients, with a mean BMI of 30.43 ± 1.07 kg/m^2^. Diabetes mellitus and smoking history were also common risk factors, each present in eight (30.8%) patients. Less common risk factors included prior sternotomy (two patients, 7.7%), chronic obstructive pulmonary disease (COPD) (one patient, 3.8%), and use of immunosuppressive medications (one patient, 3.8%).

**Table 1:** Relevant risk factors for sternal complications. Categorical variables are represented as N (%) with parametric continuous variables being represented as Mean ± SD and nonparametric continuous variables represented as Median (LQ - UQ).

Variable	Value
Number	26
Body mass index (kg/m^2^)	30.43 ± 1.07
Obese	20 (76.9%)
Diabetes mellitus	8 (30.8%)
Smoking history	8 (30.8%)
Prior sternotomy	2 (7.7%)
Chronic obstructive pulmonary disease	1 (3.8%)
Immunosuppressive medications	1 (3.8%)

Regarding other preoperative characteristics (Table 2), the mean age of the cohort was 60 years, with a male predominance (17 patients, 65.4%). Hypertension was highly prevalent, affecting 21 (80.8%) patients, followed by hyperlipidemia and coronary artery disease, each present in (15 patients, 57.7%) of the cohort. The median STS score was 0.751, and the median STS deep sternal wound infection (DSWI) score was 0.054. Other notable comorbidities included obstructive sleep apnea (nine patients, 34.6%), heart failure (six patients, 23.1%), and chronic kidney disease (six patients, 23.1%). The median CHA_2_DS_2_-VASc and HAS-BLED scores were both 2, indicating moderate risk profiles for thromboembolic and bleeding complications.

**Table 2:** Other preoperative characteristics and comorbidities. Categorical variables are represented as N (%) with parametric continuous variables being represented as Mean ± SD and nonparametric continuous variables represented as Median (LQ - UQ). STS: Society of Thoracic Surgeons, DSWI: Deep sternal wound infection, CHA_2_DS_2_-VASc: Risk score for stroke risk for patients with atrial fibrillation, HAS-BLED: Risk score for major bleeding for anticoagulated patients.

Variable	Value
Number	26
Age (y)	60 ± 2
Sex: male	17 (65.4%)
STS score	0.751 (0.527 - 0.937)
STS DSWI score	0.054 (0.044 - 0.106)
Hypertension	21 (80.8%)
Hyperlipidemia	15 (57.7%)
Coronary artery disease	15 (57.7%)
Obstructive sleep apnea	9 (34.6%)
Heart failure	6 (23.1%)
Chronic kidney disease	6 (23.1%)
Prior atrial fibrillation	4 (15.4%)
Prior percutaneous coronary intervention	4 (15.4%)
Prior myocardial infarction	3 (11.5%)
Prior Stroke	2 (7.7%)
CHA_2_DS_2_-VASc	2 (1 - 3)
HAS-BLED	2 (1 - 2)

Operative characteristics

The data presented below provide insights into the case mix and operative characteristics of patients who underwent sternal closure with the addition of aACPA, with full data in Table 3.

**Table 3:** Operative characteristics. Categorical variables are represented as N (%) with parametric continuous variables being represented as Mean ± SD and nonparametric continuous variables represented as Median (LQ - UQ). CABG: Coronary artery bypass graft.

Variable	Value
Elective	23 (88.5%)
Procedure type	
-CABG ± maze	14 (53.8%)
-Valve ± maze	10 (38.5%)
-CABG & valve ± maze	1 (3.8%)
-Aortic	1 (3.8%)
Cardiopulmonary bypass (min)	89 ± 6
Aortic cross-clamp (min)	66 ± 5
Operative time (min)	226 ± 10

**Figure 4:**
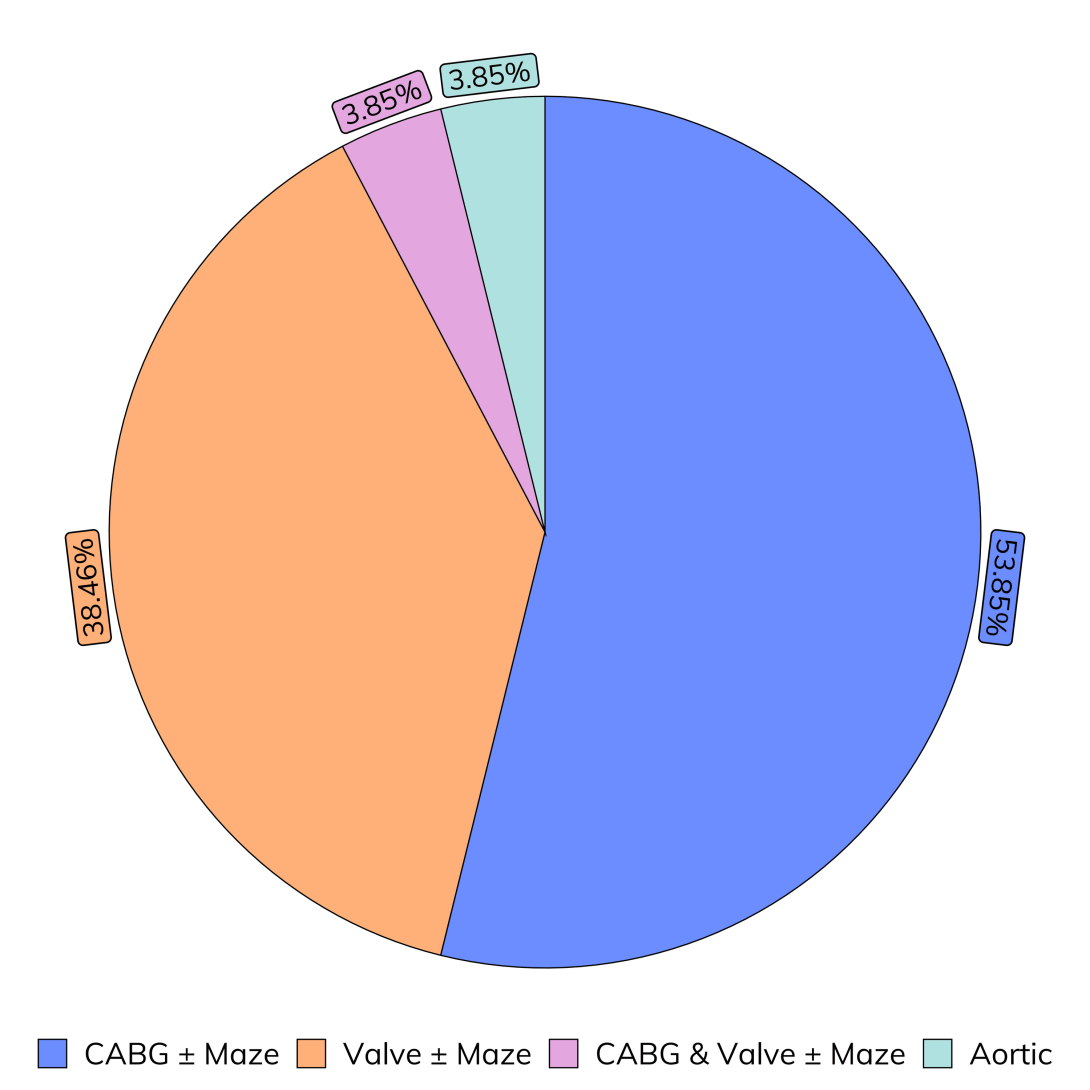
Case mix of patients that underwent sternal closure with the addition of aACPA. aACPA: Aseptically processed amnion-chorion placental allografts, CABG: Coronary artery bypass graft.

Figure 4 illustrates the distribution of procedure types. Most cases (14 patients, 53.85%) were coronary artery bypass grafting (CABG) with or without a concomitant maze procedure. Valve procedures accounted for 10 (38.46%) cases. Combined CABG and valve procedures and isolated aortic procedures were less common, with one case of each (3.85%).

**Figure 5:**
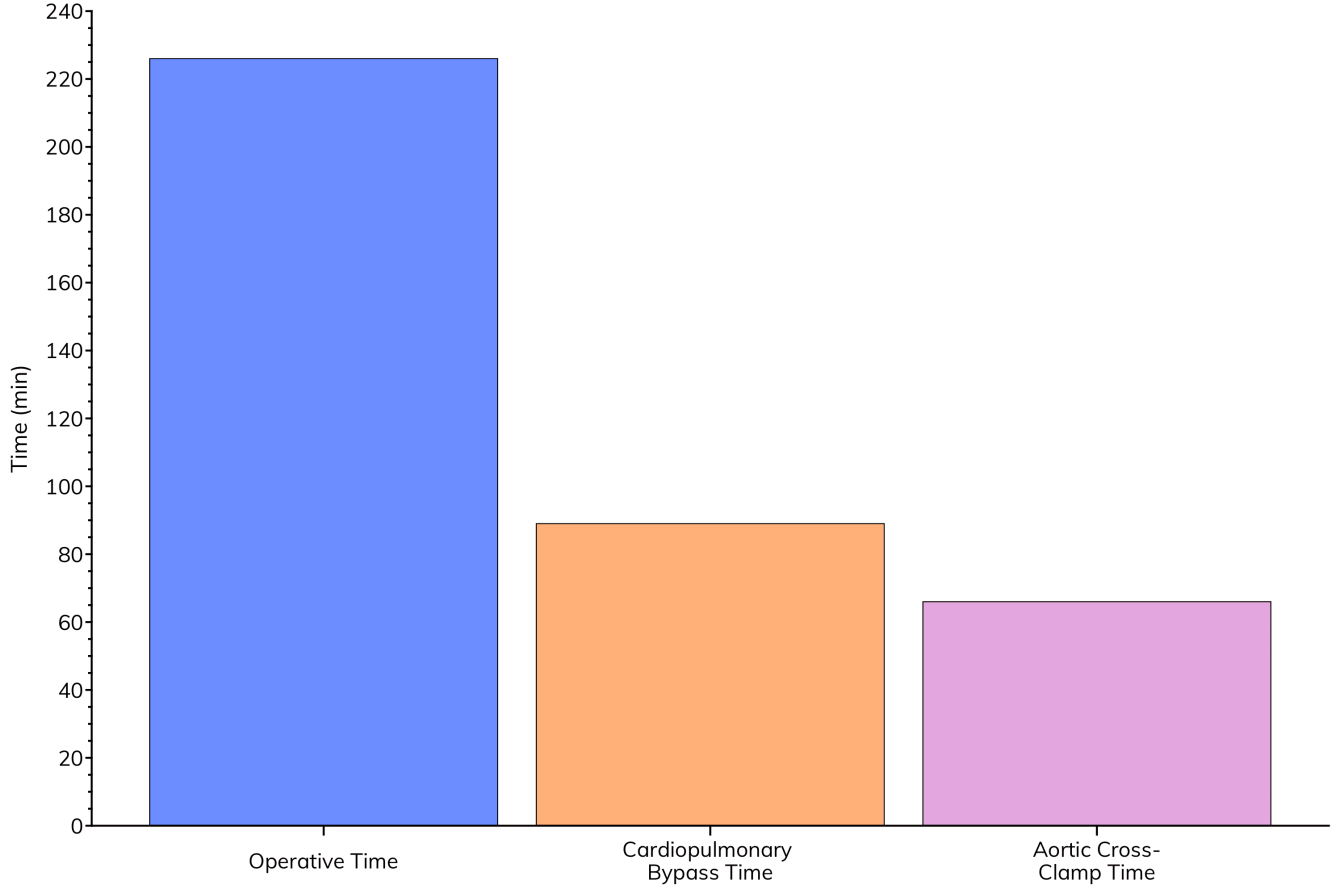
Operative times for patients that underwent sternal closure with the addition of aACPA. aACPA: Aseptically processed amnion-chorion placental allografts.

Figure 5 displays the operative times. The mean total operative time was 226 minutes. Cardiopulmonary bypass time averaged 89 minutes, while the mean aortic cross-clamp time was 66 minutes. Notably, most procedures (23 patients, 88.5%) were elective. These results indicate that the aACPA was primarily used in elective CABG and valve procedures, without substantially prolonging operative times.

Patient outcomes

The postoperative outcomes (Table 4) for patients who underwent sternal closure with aACPA demonstrate promising results. The median intensive care unit stay was three days, while the overall hospital length of stay had a median value of six days. Notably, no in-hospital mortality was reported among the cohort. In terms of complications, the data show no occurrences of sternal wound infections or dehiscence, which are significant concerns following cardiac surgery, particularly in this complex patient population, all of whom had at least one sternal risk factor. This absence of any sternal complications suggests that aACPA may support wound closure and stability. Furthermore, patient comfort appears to have been well-managed, as there were no reports of significant pain at either the 14-day or 30-day postoperative follow-up appointments. This lack of persistent pain could potentially be attributed to the stability provided by the closure technique, with better wound healing.

**Table 4:** Postoperative outcomes. Categorical variables are represented as N (%) with parametric continuous variables being represented as Mean ± SD and nonparametric continuous variables represented as Median (LQ - UQ). LOS: Length of stay.

Variable	Value
ICU LOS	3 (2 - 4)
Hospital LOS	6 (5 - 9)
Hospital death	0 (0%)
Sternal wound infection	0 (0%)
Sternal wound dehiscence	0 (0%)
Significant pain @ 14d	0 (0%)
Significant pain @ 30d	0 (0%)

These outcomes collectively suggest that the use of aACPA for sternal closure may be associated with a smooth recovery process, characterized by an absence of major sternal complications and effective pain control. The hospital and ICU stays appear to be within reasonable ranges for cardiac surgery patients, further supporting the potential benefits of this closure method in facilitating patient recovery and potentially enabling timely discharge.

**Figure 6:**
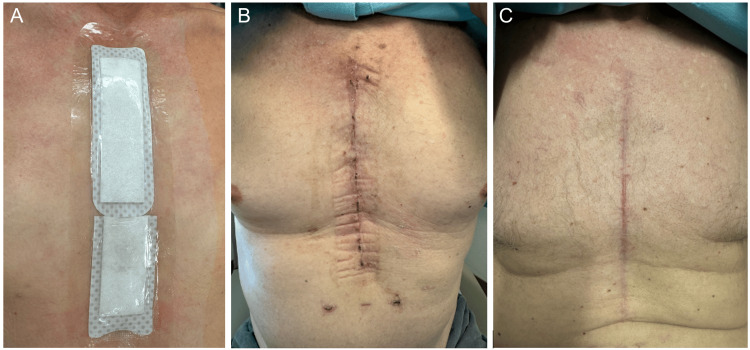
Sternal wound progression postoperatively. A: at closure in the operating room with JumpStart dressing in situ, B: two weeks postoperatively, C: four weeks postoperatively.

Figure 6 shows a representative sternal wound from our group. It shows how the use of aACPA as part of an enhanced sternal wound closure protocol, alongside suture tapes for sternal closure and an electroceutical dressing, can result in reliable, successful sternal wound closure, even in high-risk patients.

## Discussion

The use of aseptically processed amnion-chorion placental allograft (aACPA) in high-risk cardiac surgery patients undergoing median sternotomy has shown promising results in this study. Our findings suggest that aACPA may support wound closure and, therefore, reduce complications in a patient population particularly vulnerable to sternal wound issues.

Key findings and implications

Our study cohort comprised 26 patients, all of whom had at least one significant risk factor for sternal wound complications. Notably, 20 (76.9%) of the patients were obese, with a mean BMI of 30.43 kg/m^2^. Other prevalent risk factors included diabetes mellitus and smoking history, each present in eight (30.8%) of the patients. Despite this high-risk profile, we observed no instances of sternal wound infections or dehiscence at both the 14-day and 30-day postoperative follow-ups. This outcome is particularly encouraging given the typically elevated complication rates in such patient populations. The absence of significant pain reported by patients at these follow-up visits further supports the potential benefits of the aACPA use. This lack of persistent pain could be attributed to enhanced wound stability and repair.

Our operative data showed that most procedures were coronary artery bypass grafting (CABG) with or without a concomitant maze procedure, followed by valve procedures. The mean total operative time was 226 minutes, with average cardiopulmonary bypass and aortic cross-clamp times of 89 and 66 minutes, respectively. These times suggest that the incorporation of aACPA into the surgical protocol did not significantly prolong the procedures. The median intensive care unit stay of three days and overall hospital length of stay of six days are within reasonable ranges for cardiac surgery patients, especially considering the complexity of the cases. The absence of in-hospital mortality in this high-risk group further underscores the potential benefits of this approach.

Mechanism of action and relevance to high-risk patients

The efficacy of aACPA in supporting wound closure can be attributed to its unique composition. Placental tissue naturally contains a rich array of growth factors, including platelet-derived growth factor (PDGF), vascular endothelial growth factor (VEGF), and transforming growth factor-beta (TGF-β) [13]. These factors play crucial roles in various stages of wound healing, including cell proliferation, angiogenesis, and tissue remodeling [13]. PDGF is particularly important in the early stages of wound healing, promoting the proliferation and migration of fibroblasts and smooth muscle cells. VEGF, on the other hand, is a potent stimulator of angiogenesis, crucial for the formation of new blood vessels that supply oxygen and nutrients to the healing tissue. TGF-β has multiple roles, including regulating the inflammatory response, promoting the deposition of extracellular matrix, and facilitating the remodeling phase of wound healing.

Incorporating aACPA into sternal wound management may be particularly beneficial for high-risk patients, such as those with obesity, diabetes, or compromised immune systems, who often face challenges in wound healing due to impaired circulation, reduced immune function, and compromised cellular activity [14-16]. The extracellular matrix (ECM) preserved in the aACPA during aseptic processing without terminal irradiation serves as a scaffold for cell migration, proliferation, and differentiation [17]. This natural scaffold may be especially valuable in guiding tissue regeneration in patients with compromised healing capacities. The ECM in placental tissues has been shown to not only provide structural support but also interact with host cells to support their behavior, influencing processes such as adhesion, migration, and differentiation [18].

In diabetic patients, who have up to five times higher risk of developing postoperative wound infections [19], aACPA may help address multiple aspects of impaired closure The natural ECM scaffold and components can provide a supportive environment for host cell migration, revascularization, and tissue remodeling, which may be particularly beneficial given the altered ECM composition, poor vascularization, and protein imbalance in diabetic wounds [16]. For immunocompromised and elderly patients, the aACPA's ability to provide a rich source of growth factors and a supportive ECM scaffold may help compensate for the reduced healing capacity associated with weakened immune responses and age-related changes in tissue repair mechanisms [20]. In obese patients, excessive adipose tissue can impair wound healing through various mechanisms including reduced vascularization and altered inflammatory responses [14]. The natural protein composition of the aACPA may help create a more favorable environment for wound closure. The structural support provided by the ECM components of the aACPA could also be beneficial in managing the increased mechanical stress on the wound in these patients.

The combination of amnion and chorion tissues in aACPA may provide additional benefits compared to placental tissue containing solely amnion or chorion. In preclinical studies with diabetic mice, aseptically processed allograft containing both amnion and chorion was shown to support more granulation tissue formation and faster epithelialization of full-thickness wounds than amnion membrane alone [5]. This comprehensive approach to wound closure, addressing multiple aspects of tissue repair simultaneously, may explain the promising results observed in our high-risk patient cohort. By providing a supportive ECM scaffold with preserved native matrix proteins aACPA appears to create an optimal environment for sternal wound closure, even in patients with multiple risk factors for complications.

Comparison with previous research

Our findings align with and extend previous research on placental allografts in wound closure. Studies in animal models have demonstrated faster wound closure, increased granulation tissue formation, and enhanced vascularization with the use of human placental membranes. For instance, Dolivo et al. observed improved wound closure and increased granulation tissue deposition in diabetic mice treated with human placental membranes [5].

In a clinical setting, Tacktill et al. reported significant improvements in pain, function, and alignment scores, along with reduced surgical complications, in patients undergoing lower extremity soft tissue and bone reconstruction surgery using dehydrated human amnion and chorion allograft membrane, recovered and processed using the equivalent aseptic process as used with aACPA [21]. Our study extends these findings to the realm of cardiac surgery, suggesting that the benefits of aACPA may be applicable across various surgical specialties.

Financial considerations

Our analysis indicates potential cost savings associated with the use of ACPAs in sternal closure. Traditional wire closure methods, including necessary adjuncts for high-risk patients, can cost between $1,055 to $1,385 per patient. This includes expenses for sternal wires, support vests, and negative pressure wound therapy systems. In contrast, our enhanced wound closure protocol, which includes the ACPA, appears to be more cost-effective. While a formal analysis is currently ongoing, preliminary estimates suggest significant per-patient savings. This financial benefit, coupled with the potential for reduced complications and readmissions, presents a compelling argument for the adoption of ACPAs in cardiac surgical practice.

Limitations of the study

Despite the promising results, it is important to acknowledge the limitations of our study. With only 26 patients, our study has a relatively small sample size. This limits the generalizability of our findings and increases the possibility that our results could be due to chance. Our study did not include a control group undergoing traditional sternal closure without aACPA. This absence makes it difficult to definitively attribute the observed outcomes to the use of aACPA alone. All procedures were performed at a single center by one surgeon, which may limit the external validity of our findings. Practices and outcomes may vary in different healthcare settings. While our 30-day follow-up provides valuable short-term data, it does not capture potential long-term complications or benefits of the aACPA approach. While we focused on key complications like infection and dehiscence, we did not assess other potential outcomes such as patient satisfaction or quality of life measures. Lastly, the non-blinded nature of the study could have introduced bias in the assessment of subjective outcomes like pain.

## Conclusions

Increasingly complex cardiac surgery patients (older, frailer, immunocompromised, obese) face high risks of sternal wound complications, causing 20% of 30-day readmissions. This study evaluates aseptically processed, non-terminally irradiated amnion-chorion placental allograft (aACPA) for median sternotomy wound closure in high-risk patients. aACPA, derived from healthy placental tissue, contains ECM components supporting cell proliferation, angiogenesis, and tissue remodeling. Results showed no sternal wound infections, dehiscence, or significant pain at 14- and 30-day follow-ups, despite the high-risk cohort. While promising for reducing postoperative complications, larger studies with longer follow-ups are needed to confirm aACPA's efficacy and safety in cardiac surgery wound closure.
